# First Case Report of a Late Onset Knee Periprosthetic Joint Infection Caused by* Lactococcus garvieae*

**DOI:** 10.1155/2016/5053640

**Published:** 2016-10-19

**Authors:** V.-I. Neagoe, S. Zytoun, H.-J. Neuhaus

**Affiliations:** Department of Orthopaedics and Traumatology, St. Vincenz-Hospital, 58706 Menden, Germany

## Abstract

*Lactococcus garvieae* is known as a Gram-positive, catalase-negative, and facultatively anaerobic fish pathogen. The association between* Lactococcus *spp. and human infectious diseases is described as being mainly associated with lumbar osteomyelitis, hepatic abscess, and infective endocarditis. In the literature of orthopedic post-prosthetic infections,* L. garvieae* was associated with a case of hip prosthetic infection in a fishmonger woman. We present the case of a 79-year-old male patient with multiple comorbidities, who is admitted to our center with a 5-day history of pain, swelling, and motility disorder of the right knee by the presence of a bicondylar knee replacement surgery, which was performed due to gonarthrosis 17 years ago. The radiographies of the right knee revealed no signs of displacement or loosening of the prothesis. After multiple radical debridements including VAC therapy and targeted antibiotic therapy we have managed to defeat the infection without exchange arthroplasty. Although we could not demonstrate the source of infection, we can only presume that in our case the source of infection was represented by the ingestion of possibly contaminated food. The patient had a habit of eating Nile perch fish (*Lates niloticus*) every 4 weeks. We illustrated once more the possibility of a late onset* L. garvieae* related orthopedic periprosthetic joint infection by multiple comorbidities.

## 1. Introduction

*Lactococcus garvieae* is known as a Gram-positive, catalase-negative, and facultatively anaerobic fish pathogen. It was first considered to be included in* Streptococcus genus*, from which it was separated in 1985 [1]. Originally described as* Streptococcus garvieae* and being isolated from cases of bovine mastitis,* L. garvieae* is nowadays frequently misidentified as belonging to the* Enterococcus species* [2]. More precisely,* L. garvieae* is frequently confused with* Enterococcus faecalis*, because of their morphological similarities [2, 3]. Both germs are negative for catalase, arabinose, and raffinose while being positive for maltose, fructose, and mannitol [4, 5]. The fermentation of sorbitol makes the differentiation of the two germs feasible, which is negative by* Lactococcus *spp. and positive by* Enterococcus faecalis*. In the literature, the association between* Lactococcus *spp. and human infectious diseases is described as being mainly associated to lumbar osteomyelitis, hepatic abscess, and infective endocarditis [6, 7]. More recently, in orthopedic post-prosthetic infections,* L. garvieae* was associated with a case of hip prosthetic infection in a fishmonger woman [8]. A defect in the anatomy or physiology of the gastrointestinal tract (gastric ulcer, colonic diverticulum, or prior surgery) and the use of acid-suppressing medications may play a key role in discovering the pathogenesis of the disputed coccus [9–11].

## 2. Case Report

We present the case of a 79-year-old male patient, who is admitted to our center for endoprosthetic orthopedic surgery of St. Vincenz-Hospital, Menden, Germany. The patient is admitted with a 5-day history of pain, swelling, and motility disorder of the right knee. The medical history of the patient included polyarthritis rheumatica, obstructive sleep apnea syndrome, pacemaker implantation (Biotronik Talos DR) in 2005 for atrial fibrillation, condition after angioplasty for coronary disease (2007), degenerative disc disease, obesity, antral gastritis, endoscopic Zenker's diverticulum repair performed in 2014, and uncontrolled type 2 diabetes. The surgical history of the patient included sigma resection for complicated diverticulitis with hemorrhage in 2004 and the substrate of our case represented by the presence of a right bicondylar knee replacement surgery, which was performed due to gonarthrosis 17 years ago. On clinical examination we suspected a local infection due to the presence of the Celsian clinical features for acute inflammation, represented by pain, swelling, local heat, erythema, and movement disorder with partial loss of function (functio laesa). The radiographies of the right knee revealed no signs of displacement, loosening of the prothesis, or other pathological signs which would include unsealing, fracture, or cortical bone modifications. Without the presence of SIRS (Systemic Inflammatory Response Syndrome) that collection of blood cultures is unnecessary and we decided to aspirate the knee joint. A seropurulent fluid is extracted and sent for microbiological examination. A Bruker MALDI-TOF Biotyper was used and the result showed us the presence of isolated* Lactococcus garvieae*, which according to the antibiogram showed susceptibility to Erythromycin, Cefazolin, Cefuroxime, Cefotaxime, Imipenem, Meropenem, Ertapenem, and Linezolid. Correlating our findings, clinically, microbiologically, and with the pathological modified laboratory data, which included a leukocyte count of 10.4/nL (normal range: 4.0–10.0), creatinine: 1.4 mg/dL (normal range: 0.00–1.20), and a C-reactive protein of 12.5 mg/dL (normal range < 0.50), we decided to institute an intravenous therapy with Cefazolin 2 g for every 8 hours. After 6 days without a remarkably improvement of the symptoms or of the joint motility, we performed an exploratory arthroscopy of the affected joint. During the arthroscopic lavage, partial synovectomy was performed. Furthermore 4 samples have been taken and sent for culture and microbiological examinations. For the postoperative secretion control we placed 2 Redon drains in the knee joint. The microbiological report from the medial synovial membrane showed no bacterial growth, but the examination of the sample taken from the upper recess of the knee and from the synovial fluid confirmed once again the infection with* Lactococcus garvieae*. After 11 more days of Cefazolin therapy, the laboratory findings have shown a satisfactory decrease of the C-reactive protein to a value of 2.92 mg/dL, contrary to the clinical findings, which showed the persistence of swelling, pain, and motility disorder. Based on the constellation of the clinical and laboratory findings we decided that an open revision surgery of the knee is rational. The intraoperative findings revealed a partially delaminated inlay which required no urgent replacement and an inflamed synovia. The quadriceps and patellar tendons were intact and had no role in the etiology of the motility disorder of the knee joint. Under these circumstances we decided to take samples again, perform a synovial resection, jet lavage joint cleansing, and debridement, and use a vacuum-assisted negative pressure wound closure therapy system (V.A.C-Ulta™ Therapy). The microbiological analyses of the samples have attested once more the presence of* Lactococcus garvieae* and a third surgical procedure was performed 6 days later in the context of revision surgery. During the procedure we did probe preservation, joint lavage, and the change of the V.A.C-Ulta system. During this period of time the patient had received without disruption the initial intravenous antibiotic therapy with Cefazolin. The latest microbiological findings showed no growth of* Lactococcus garvieae* and we decided after 10 days from the last surgery to remove the V.A.C-Ulta system and to perform probe preservation, drainage, and secondary wound closure. After 2 days we removed the drains and sent the intra-articular part of the Redon tubes for microbiological analysis. The results were negative for infection and after physiotherapy combined with continuous passive motion (CPM) the patient managed to improve his right knee range of motion to 0/0/95 degrees. After 44 days of therapy (24 days with Cefazolin antibiotic therapy) we managed to discharge the patient with no signs of infection and with satisfactory joint mobility. At the follow-up controls at 2, 4, and 6 weeks after discharge, evidences of recurrence infection were not present. With further unaffected joint motility, our patient remains satisfied with the therapeutic outcomes.

## 3. Discussion

*L*.* garvieae* is rarely associated with human infections; it is a rare human pathogen with low virulence in humans but can be highly active, causing septicemia in many fish species (prawns, rainbow trout, yellow tail, and gray mullet), and also can cause mastitis in ruminants [4, 12, 13]. As other authors do, we also presume that in the majority of cases the source of infection is represented by the ingestion of contaminated food, especially freshwater fish species [14].* L. garvieae* is more known to be associated with infective endocarditis in patients with colonic diverticulitis as with orthopedic periprosthetic infections. In our presented case, the preoperative cardiovascular examination and pacemaker check-up showed no suspicion for endocarditis. Regarding diverticulitis, as mentioned above, our patient is known as having a history of sigma resection for complicated diverticulitis and endoscopic Zenker's diverticulum repair. Having no gastrointestinal symptoms, gastroscopy and colonoscopy were not considered mandatory in the context of a joint infection. A gastroscopy or colonoscopy would make sense as in the case of* L. garvieae* infective endocarditis affecting a patient with known colonic diverticulitis who got mitral valve repair with autologous pericardium as described lately in Italy in 2012 [15]. A short review of the literature shows a connection between* L. garvieae* and reported cases of bacteremia [9], osteomyelitis [6], liver abscess [7], and peritonitis [9]. Regarding the orthopedic periprosthetic joint infections,* L. garvieae* has represented in only one case the etiology of hip prosthetic joint infection in a fishmonger woman; the patient was under immunosuppression therapy and lived near the sea and consumed frequently fish and shellfish. In that case a two-stage exchange arthroplasty was planned, the first stage being represented by the removal of the infected prosthesis and insertion of a gentamicin spacer for 6 weeks [8]. In our case, following the recommendation of the 2013* Proceedings of the International Consensus Meeting on Periprosthetic Joint Infection from Philadelphia* [16], after multiple radical debridements including negative wound pressure therapy and targeted intravenous antibiotic therapy we have managed to defeat the infection without exchange arthroplasty or inlay replacement. Analysing previous studies related to the pathogenesis of* L. garvieae*, cardiovascular comorbidities, immunosuppression, and colonic diverticulosis seemed to play a key role in the* L. garvieae* related infections.

Our patient also has a history of cardiovascular comorbidities, arthritis, uncontrolled diabetes type 2, and obesity which altogether contribute to affecting the immune status of the patient. Without finding a portal of entry or demonstrating the source of infection, we can only suppose that lately the patient having a habit of eating Nile perch (*Lates niloticus*) has probably eaten possibly contaminated Nile perch. Although this source of infection is also in the literature described, in our case the dissemination of* L. garvieae* remains at last uncertain.

## 4. Conclusion

*L*.* garvieae*, highly virulent in zoonotic diseases, is a rare human pathogen and its diagnosis is often difficult especially when the bacteriologist is not aware of the possible connection with contaminated food or animal contact. In our presented case, despite our investigations, the source and the pathogenesis of the infection were not established. Nevertheless, we only assume, like other authors also do, that the handling and consumption of contaminated fish can play a role in this sort of infections. Our case illustrates once more the possibility of a late onset* L. garvieae* related periprosthetic joint infection by a patient with multiple comorbidities. Although guiding us after the* Proceedings of the International Consensus Meeting on Periprosthetic Joint Infection* from 2013, as a particularity of our case, the removal of the prosthesis was not necessary. In our opinion treating rare periprosthetic joint infection remains furthermore a challenge, and in the absence of a specific guideline regarding rare joint infections we believe that clinicians should exercise their instinctive clinical aptitude and wisdom in making decisions related to each and every patient individually.
